# Genomic analysis of matrix metalloproteinases affecting the prognosis and immunogenic profile of gastric cancer

**DOI:** 10.3389/fgene.2023.1128088

**Published:** 2023-04-18

**Authors:** Chaofeng Yuan, Jialin Yuan, Huijie Xiao, Haitao Li, Yang Jiang, Rongnan Zhai, Jinjing Zhai, Hua Xing, Jiannan Huang

**Affiliations:** ^1^ Department of Gastrointestinal Colorectal Surgery, China-Japan Union Hospital of Jilin University, Changchun, China; ^2^ Department of Breast Surgery, China-Japan Union Hospital of Jilin University, Changchun, China; ^3^ Department of Orthopedics, China-Japan Union Hospital of Jilin University, Changchun, China

**Keywords:** gastric cancer, MMP-matrix metalloproteinase, immunogenic profile, clinical diagnosis, immunotherapy, clinical treatment, genomics

## Abstract

This study systematically and comprehensively analyzed the characteristics of matrix metalloproteinases (MMPs) in gastric cancer (GC) and revealed the relationship between MMPs and prognoses, clinicopathological features, tumor microenvironment, gene mutations, and drug therapy response in patients with GC. Based on the mRNA expression profiles of 45 MMP-related genes in GC, we established a model that classified GC patients into three groups based on cluster analysis of the mRNA expression profiles. The 3 groups of GC patients showed significantly different prognoses as well as tumor microenvironmental characteristics. Next, we used Boruta’s algorithm and PCA method to establish an MMP scoring system and found that lower MMP scores were associated with better prognoses, lower clinical stages, better immune cell infiltration, lower degrees of immune dysfunction and rejection, and more genetic mutations. Whereas a high MMP score was the opposite. These observations were further validated with data from other datasets, showing the robustness of our MMP scoring system. Overall, MMP could be involved in the tumor microenvironment (TME), clinical features, and prognosis of GC. An in-depth study of MMP patterns can better understand the indispensable role of MMP in the development of GC and reasonably assess the survival prognosis, clinicopathological features, and drug efficacy of different patients, thus providing clinicians with a broader vision of GC progression and treatment.

## Introduction

Due to the improvement of general hygiene and wide applications of *H. pylori* quadruple therapy and gastroscopy, the incidence and mortality of gastric cancer (GC) have declined significantly worldwide in recent years (Choi et al., 2018). However, it is still a challenging disease in many countries and regions. The incidence of GC ranks third after lung and liver cancers, with a mortality rate of 8.2% (Bray et al., 2018). Moreover, the age of onset for GC has been decreasing in the last decades (Zhou et al., 2016). The development of GC is a complex process involving numerous factors (such as genetic and environmental factors) and steps. Genetic factors are supposed to be the most important factor for GC development. For instance, in a Swedish study that included 23,386 twins, it was found that if one of the twins was diagnosed with GC, the other was five times more likely to develop GC (Ekstrom et al., 2000), suggesting the importance of genetic factors in the development of GC. Previous studies have identified many susceptibility genes associated with GC. Cho et al. found that high mRNA expression of *CTNNBA1*, *TOP2A*, *LZTR1*, *EXOSC3*, *LBA1,* and *CCL5* were closely associated with worse prognoses of GC (Cho et al., 2011). Kang et al. found that mRNA expression of *ESRRG* was significantly reduced in GC and negatively correlated with the stage and prognosis of GC (Kang et al., 2018). However, the prediction accuracy based on these genes is not satisfied. There are some targeted therapies, such as trastuzumab for HER-2, bevacizumab for VEGF-A (Wadhwa et al., 2013), and cetuximab for EGFR, that are currently widely used. However, only 5%–10% of GC patients are HER-2 positive, half of the GC patients have mutant KRAS genes (Albertini et al., 2017) and not all patients respond well to bevacizumab. All these indicate that more genes that can better reflect the severity and evaluate the prognosis of GC need to be identified (Wu et al., 2014; Ciliberto et al., 2015).

MMPs, belonging to the family of Ca2^+^ and Zn2^+^ dependent protein hydrolases, are synthesized and secreted by a variety of cells and can degrade components in the extracellular matrix (ECM). MMPs play important roles in cell proliferation, differentiation, migration, and apoptosis. There are 26 members in the MMP family, which can be further divided into five subgroups (Scherer et al., 2008). MMP2 and MMP9 are rich in invasive pseudopods and therefore have a strong catabolic effect on ECM both *in vitro* and *in vivo* (Murphy and Courtneidge, 2011). During tumor development, MMP2 and MMP9 may be involved in the regulation of tumor angiogenesis by undermining immunity, activating the TGF-β signaling, and releasing VEGF and bFGF (Solov'Eva et al., 2014), thus promoting the rapid growth and distant metastasis of tumor cells. Therefore, MMP may act as a bond between tumor cells and the tumor microenvironment of GC. In a previous meta-analysis, it was found that high expression of MMP2 was significantly associated with a worse prognosis of GC (Wang et al., 2014a). In another large-scale study, strong associations were observed between the positive expression of MT1-MMP, and peritoneal metastasis, lymph node metastasis, and worse prognosis of GC (Mimori et al., 2008). These previous studies indicate that MMPs might be important diagnostic and prognostic markers for GC. Therefore, analysis of MMP-related genes might reveal insights for the diagnosis and prognosis prediction of GC.

Thus, in this study, we established a molecular model that can classify GC patients into 3 patterns based on the mRNA expression of MMPs, which showed different prognostic and tumor microenvironmental features. Furthermore, based on this molecular model, the establishment of an MMP scoring system might be valuable for early diagnosis, prognosis prediction, and treatment of patients with GC.

## Results

### Establishment of a model based on the expression profiles of MMP-related genes

**FIGURE 1:**
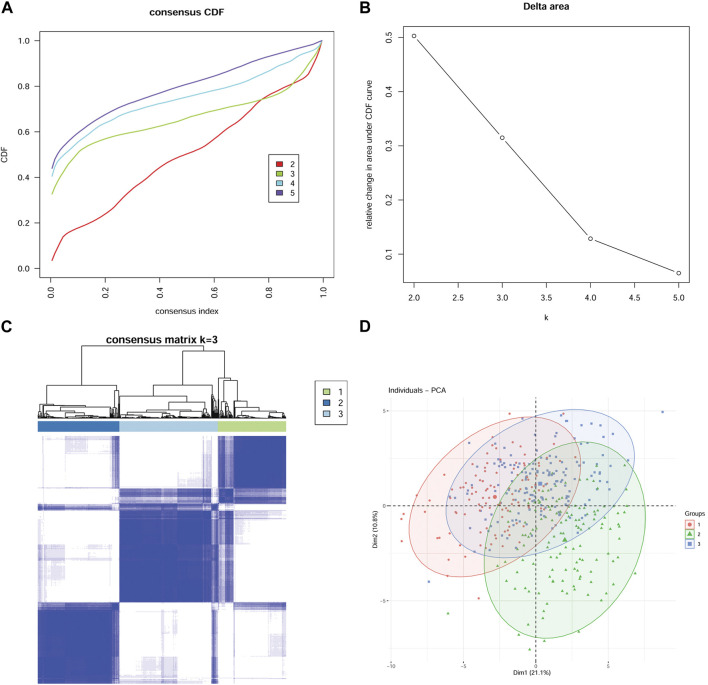
Establishment of a model based on expression profiles of 45 MMP-related genes Consensus CDF Plot **(A)**: A plot of the cumulative distribution function for different values of K, used to determine the value of K at which the CDF reaches its approximate maximum. Thus the smaller the slope of the curve, the better the K value. Delta Area Plot **(B)** demonstrates the relative change in the area under the CDF curve when K is compared to K-1. The smaller the relative change, the higher the confidence level of the clustering. Therefore, after combining the Consensus CDF Plot and Delta Area Plot, K = 3 is the optimal number of clusters. Consensus Matrix Plot **(C)** verifies the high clustering confidence at K = 3. The higher the clustering confidence and differentiation, the higher the degree of color aggregation of different matrices in the matrix plot and the more obvious the degree of discrepancy between matrices. **(D)** The principal component analysis (PCA) algorithm confirmed the validity and reasonableness of the classification.

In this study, mRNA expression data of 45 MMP-related genes in GC were selected and analyzed. To maximize the classification of models with different characteristics, we applied an unsupervised cluster analysis method. The consensus cluster analysis revealed the optimal K = 3 (Figures 1A–C). As a result, the 411 GC patients were divided into 3 groups, i.e., Cluster (Group) 1, Cluster (Group) 2, and Cluster (Group) 3 (C1 = 113, C2 = 135, C3 = 163). The distribution of the 3 groups based on mRNA expression downscaled and validated by the PCA algorithm is shown in Figure 1D.

### The three MMP- related groups are different in prognosis and tumor microenvironmental characteristics

**FIGURE 2:**
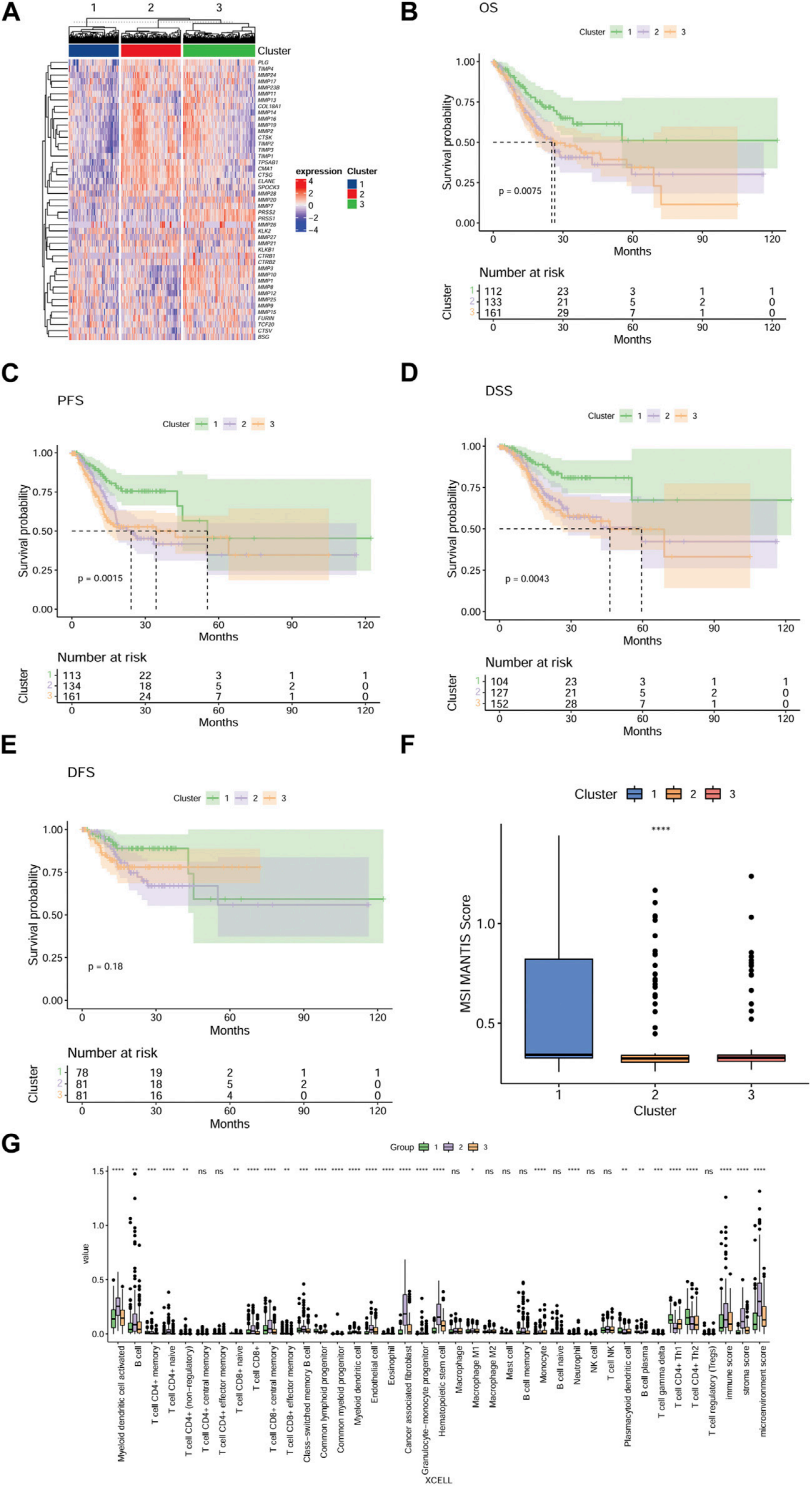
The three MMP-related groups in GC patients have significant differences in prognosis and tumor microenvironment characteristics **(A)** A heat map shows the expression levels of 45 MMP-related genes in three groups, and each row in the heat map represents one MMP-related gene. The degree of expression of the gene varies among the three groups, the higher the expression level, the redder the color, and the lower the bluer. Kaplan-Meier survival analysis comparing the differences in OS **(B)**, PFS **(C)**, DSS **(D)**, and DFS **(E)** among the three groups of patients. **(F)** Box plots depict the MSI scores of the three groups of patients. **(G)** Box plots showing differences in the tumor microenvironment among the three groups of patients. *p < 0.05; **p < 0.01; ***p < 0.001; ****p < 0.0001. Ns, not significant.

Aiming at directly comparing the expression levels of the 45 MMP-related genes in the three groups, a heat map is drawn and the results are shown in Figure 2A. To compare the prognosis of patients in different model groups, we used Cox regression to analyze the survival time of patients in these three groups and found that there were significant differences in overall survival (OS) (*p* = 0.075; Figure 2B), progression-free survival (PFS) (*p* = 0.0015; Figure 2C), and disease-specific survival (DSS) (*p* = 0.0043; Figure 2D) among the three groups of patients. More specifically, patients in group C1 have better prognoses than patients in groups C2 and C3. However, disease-free survival (DFS) was not significantly different between these three groups (*p* = 0.18; Figure 2E). MSI scores are criteria that reflect the instability level of the microsatellite. To observe the microsatellite instability and predict the immunotherapy efficacy of patients in three groups, we further calculated and compared the MSI scores, and found that MSI scores were significantly different among the three groups (*p* < 0.0001 for all; Figure 2F). Specifically, MSI scores are higher in the C1 group than in C2 and C3 groups. Furthermore, to evaluate the correlation between the TME and those three groups, we calculated the immune score, matrix score, and microenvironment score using XCELL, and analyzed their difference. As shown in Figure 2G, the immune cell infiltration in the three groups, indicated by activated myeloid dendritic cells, T lymphocytes, endothelial cells, hematopoietic stem cells, tumor-derived fibroblasts, neutrophils, monocytes infiltration and immune scores, stromal scores, and microenvironment scores, is different in the three groups. These results indicated that our model using these 45 MMP-related genes could effectively classify GC patients into different groups with different immunologic and prognostic features.

### Establishment of an MMP scoring system for GC patients

**FIGURE 3:**
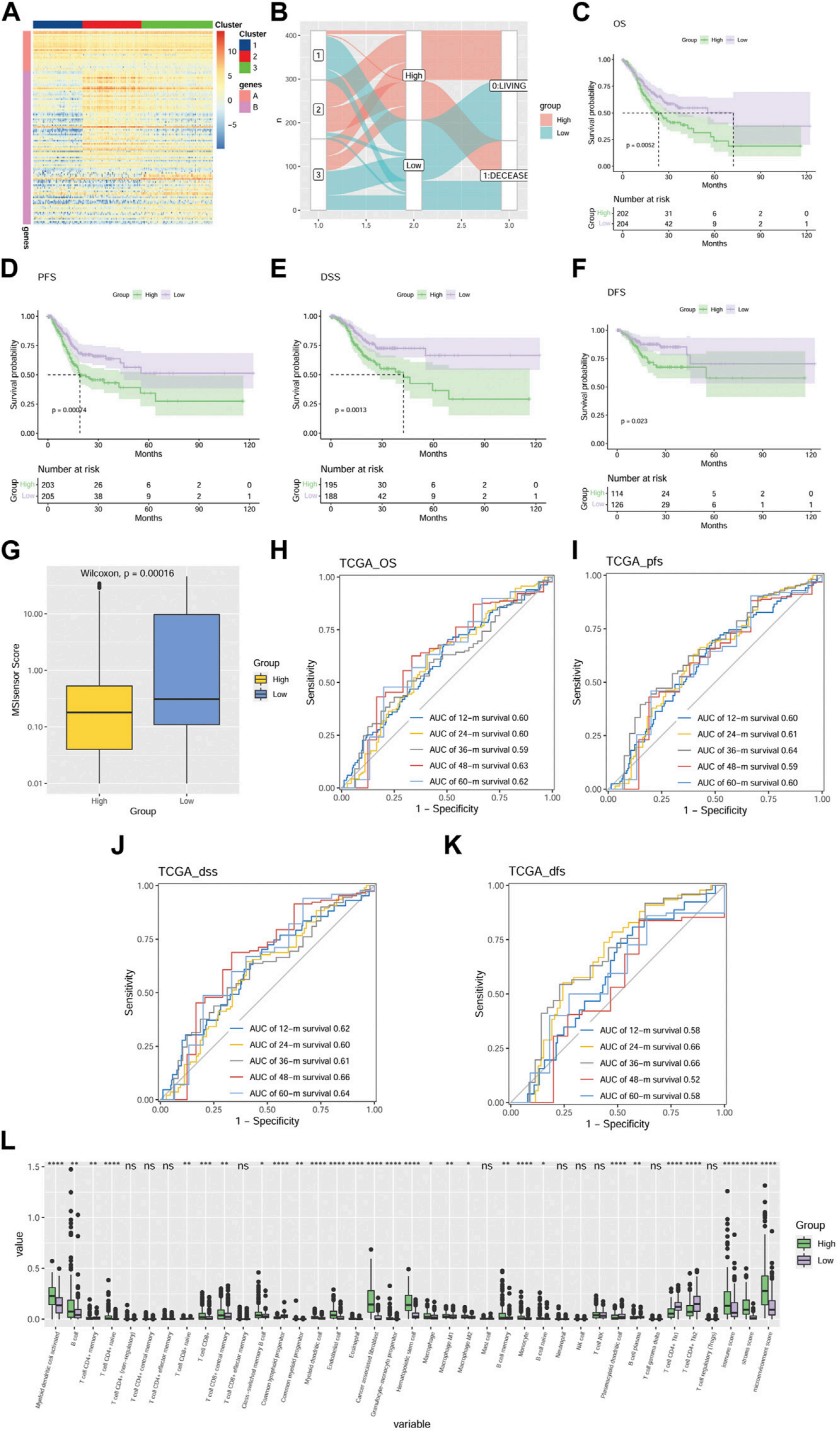
Development of an MMP scoring system for GC patients **(A)** Heat map depicts the expression situation of the 126 most abundant DEGs in the genome in three different gene clusters. Those DEGs are divided into Signature A and B genes. Each row represents one DEG. The higher the expression level of a DEG, the redder the color, and the lower the bluer. **(B)** Alluvial plots depicting the relationship between three molecular models, MMP scores, and survival status. Kaplan-Meier survival analysis comparing the differences in OS **(C)**, PFS **(D)**, DSS **(E)**, and DFS **(F)** among the three groups. **(G)** Box plots show the difference in MSI scores between the two groups. ROC is based on MMP scores for OS **(H)**, PFS **(I)**, DSS **(J)**, and DFS **(K)**. **(L)** Box plot compares the discrepancy of immune infiltration level of various immune cells and immune score, stromal score, and microenvironmental score in TME between two groups of patients with MMP scores. The higher the immune infiltration and the immune score, stromal score, and microenvironmental score, the higher the value. *p < 0.05; **p < 0.01; ***p < 0.001; ****p < 0.0001. Ns, not significant.

To downscale the prognosis and the differences in TME of different patients, we established the MMP scoring system to make the subsequent clinical analysis more concise and clearer. We identified the DEGs among the three groups using the Boruta algorithm and obtained the signature A genes and signature B genes. A heat map was depicted to directly visualize the expression level of the 126 most abundant DEGs among the three groups (Figure 3A). All GC patients were then scored using the principal component analysis (PCA) algorithm. According to the median of the scores, GC patients were divided into a high MMP score group and a low MMP score group. To delineate the correlation between the MMP score, the survival status of the patients, and the MMP-related groups, Figure 3B was depicted. As the result, the patients in the C1 group with the best prognosis are those with low MMP scores, while the patients in the C2 and C3 groups with relatively poor prognosis are mostly those with high MMP scores. Aiming at further analyzing the relationship between MMP scores and prognosis, the results are shown in Figures 3C–F. Low MMP scores are significantly associated with OS (*p* = 0.0052), PFS (*p* = 0.00074), DSS (*p* = 0.0013), and DFS (*p* = 0.023). For the sake of identifying the difference in microsatellite instability and predicting the efficacy of immunotherapy, we analyzed the MSI scores in different MMP patterns. We showed that the difference in MSI scores was significant between the two groups (*p* < 0.0001; Figure 3G), consistent with those from the C1, C2, and C3 groups. That is, the low MMP score group with a good survival outcome had a higher MSI score as did the C1 group. In the case of analyzing and validating the predictive efficacy of the MMP scoring system, we further calculated the AUC of our model in predicting OS, PFS, DSS, and DFS (Figures 3H–K), and showed that MMP scores are effective in predicting survival at 12, 24, 36, 48 and 60 months in patients with GC. To compare and identify the discrepancy in TME of different MMP scoring groups, again we calculated the infiltration ratios of multiple immune cells, the immune score, the stromal score, and the microenvironmental score using Xcell (Figure 3L). We showed that lower MMP scores were associated with lower infiltration of myeloid dendritic cells, B lymphocytes, cytotoxic T lymphocytes, monocytes, macrophages, tumor-derived fibroblasts, and lower immune, stromal, and microenvironment scores, indicating an inextricable relationship between MMP score and tumor microenvironment.

### Association of MMP scores with clinicopathological features in patients GC

**FIGURE 4:**
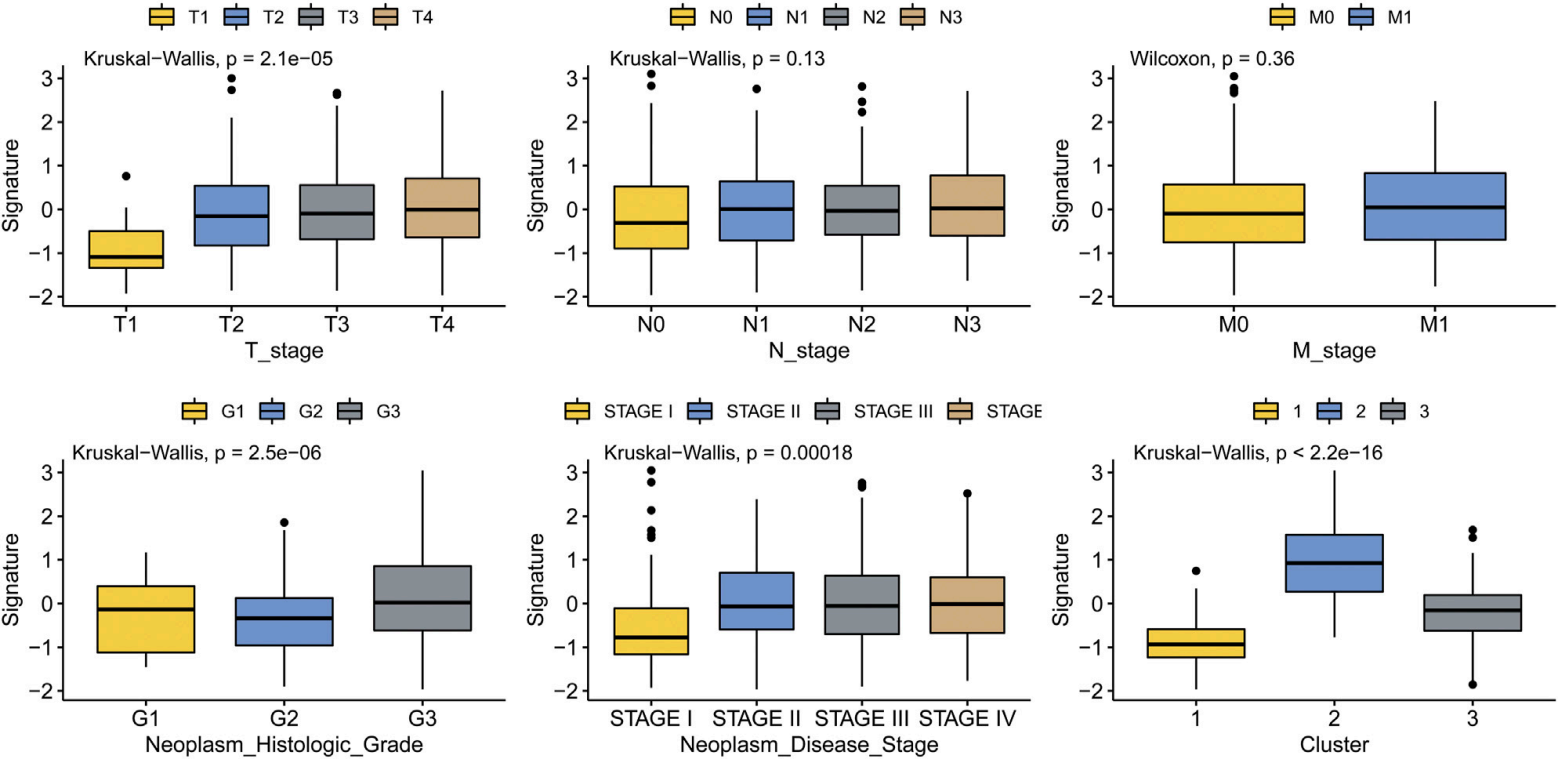
Association of MMP scores with clinicopathological features in patients with gastric cancer.

To analyze the correlation between MMP scores and major clinicopathological features in patients with GC and validate the clinical value of the MMP scoring system, we delineate the box plots. As shown in Figure 4, all the clinicopathological features, except for the N stage and M stage, are associated with the MMP scores. The MMP scores were significantly lower in patients in stage T1 than those in stages T2, T3, and T4, significantly higher in patients with G3 than those with G1 and G2, significantly lower in patients in stage I than those in stages II, III and IV, significantly lower in patients in group C1 than those in groups C2 and C3. These results indicate that MMP scores are closely related to major clinicopathological characteristics of gastric cancer.

### Gene set enrichment analysis of DEGs

**FIGURE 5:**
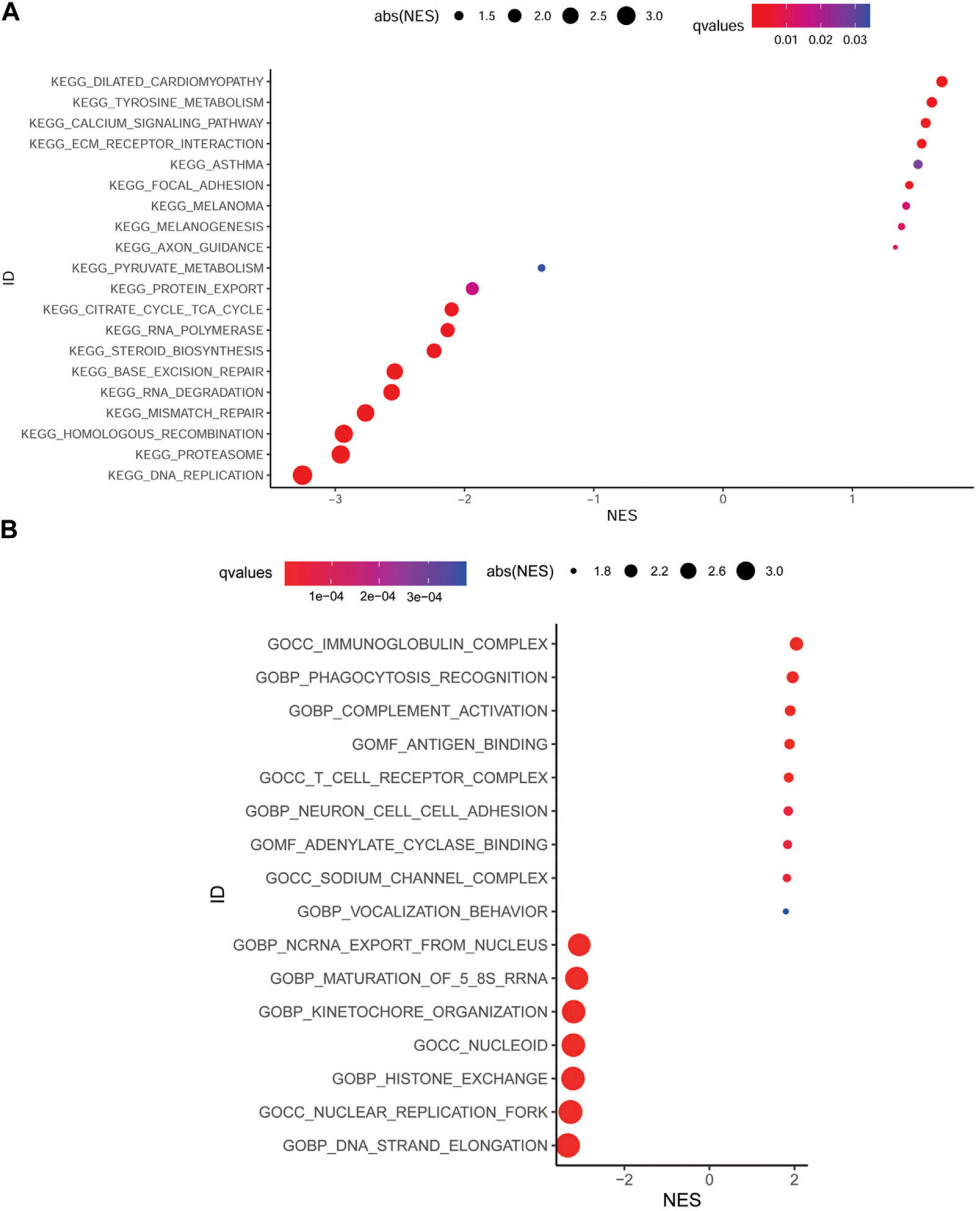
Bubble plots depicting the enrichment of KEGG **(A)** and GO **(B)** in different score groups of MMP.

Aiming at further understanding the terms (i.e., biochemical processes, molecular functions, and cellular components), signaling pathways involved in the low MMP score and high MMP score groups and speculating the roles they play in the development of GC, we performed KEGG and GO enrichment analysis of the DEGs using gene set enrichment analysis (GESA); significantly enriched terms or pathways are illustrated using the bubble plots in Figures 5A, B. KEGG enrichment analysis showed that various signaling pathways, including DNA replication, proteasome, homologous recombination, and mismatch repair, were enriched in the low MMP score group. While various pathways, including dilated cardiomyopathy, tyrosine metabolism, calcium signaling pathway, and ECM receptor interaction, were significantly enriched in the high MMP score group. GO enrichment analysis demonstrated that DNA strand elongation, nuclear replication fork, histone exchange, and nucleoid and kinetochore organization terms were enriched in the low MMP score group, whereas the immunoglobulin complex, phagocytosis recognition, complement activation, and antigen binding terms were enriched in the high MMP score group.

### Gene mutations in different MMP score groups

**FIGURE 6:**
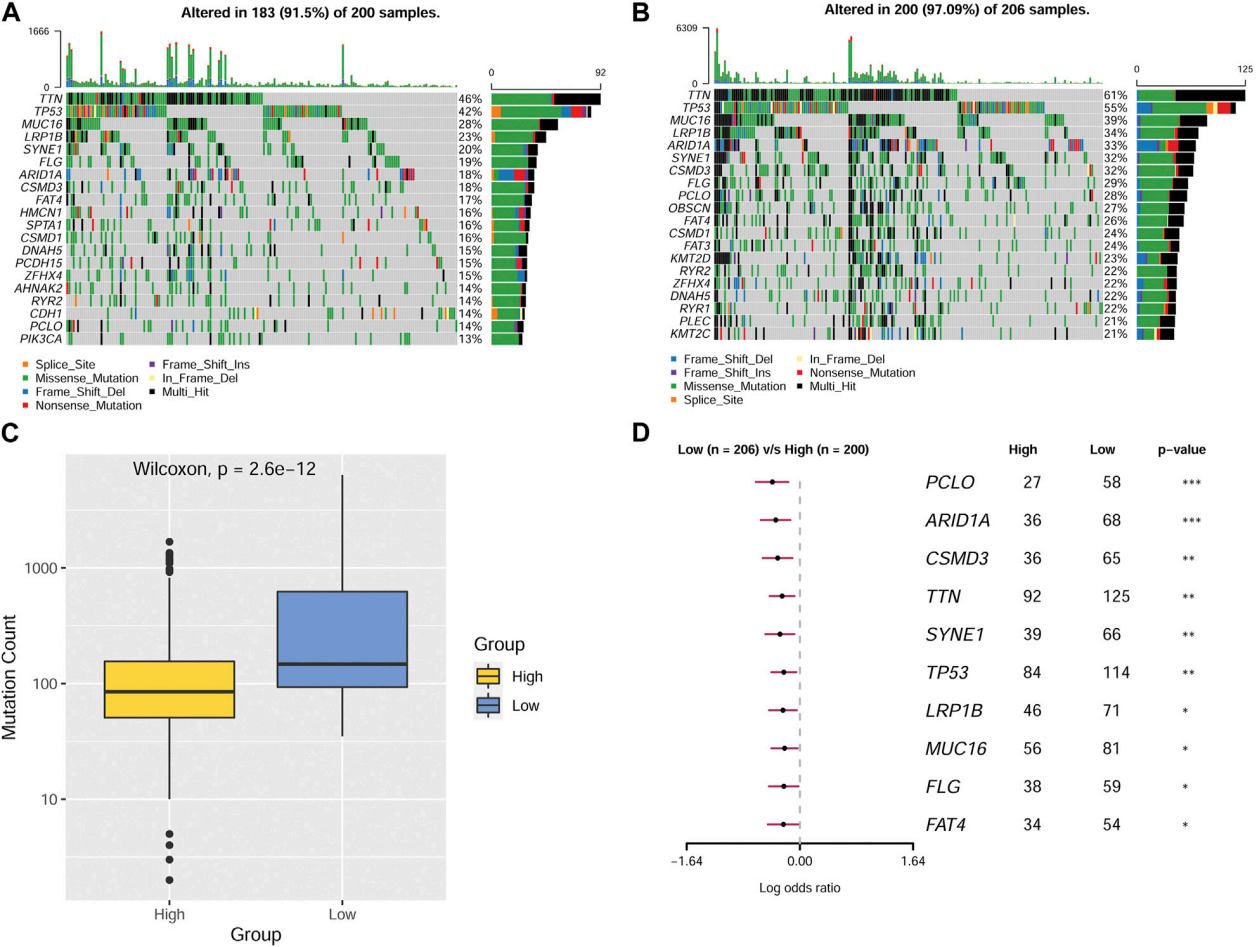
Mutations in patients from different MMP scoring groups **(A)** Mutations in MMP high-scoring group. **(B)**Mutations in MMP low-scoring group. **(C)** Mutation counts in different MMP scoring groups. **(D)** Forest plot depicting statistical differences of various genes in different MMP scoring groups.

A gene mutation is a type of contributing factor in the development of GC, different gene mutations might play different roles. For the sake of further identifying the role of different genes on gastric cancer, we analyze and compare gene mutations between patients in the two MMP score groups. The data were presented as gene mutation maps as shown in Figures 6A, B. The highest mutation rates were found in *TTN*, *TP53*, *MUC16,* and *LRP1B* genes, which were present in both the high and low MMP score groups. Specifically, the four genes in the low MMP score group had higher mutation rates than those in the high MMP score group. Moreover, to vividly compare the overall gene mutation counts in these two MMP score groups, we drew a box plot that delineates that the low MMP score group has higher overall mutation counts than the high MMP score group (Figure 6C). To verify whether the mutation counts in different genes were statistically significant between the two MMP score groups, we applied the logistic binary regression analysis. As shown in Figure 6D, we found that the high MMP score group tends to have fewer mutation genes.

### MMP scores and drug treatment

**FIGURE 7:**
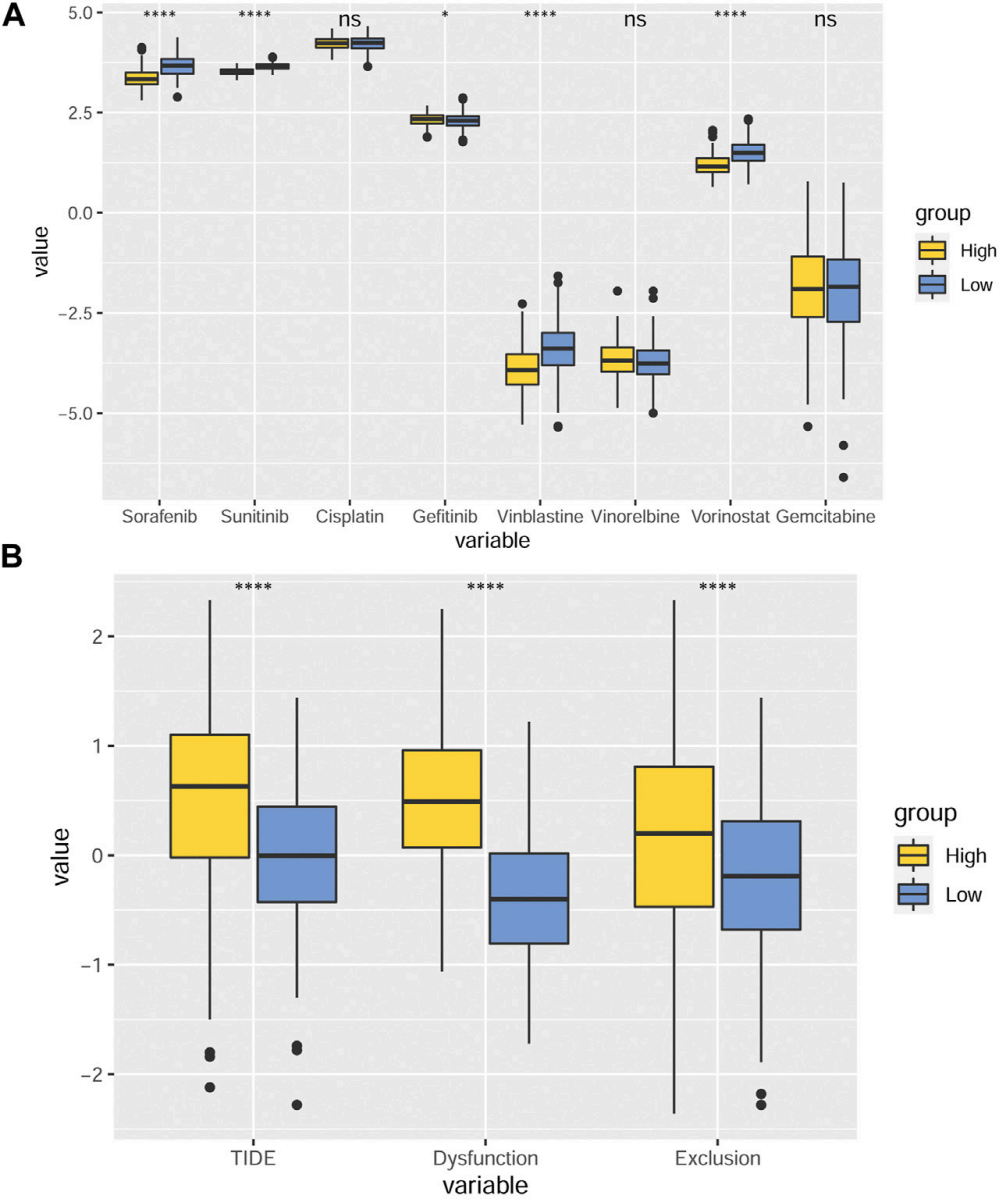
MMP scores and drug IC50 inference **(A)** Box plots depicting the differences in predicted IC50 values for sorafenib, sunitinib, cisplatin, gefitinib, vincristine, vorinostat, and gemcitabine between the different MMP scoring groups. **(B)** Differences in TIDE scores, immune dysfunction, and rejection in different MMP scoring groups. *p < 0.05; **p < 0.01; ***p < 0.001; ****p < 0.0001. Ns, not significant.

Drug therapy plays an important role in the clinical diagnosis and treatment of GC. Aiming at finding out the efficacy and sensitivity of various chemotherapy and targeted therapy drugs on GC, we compared the predicted IC50 values of eight different drugs, including sorafenib, sunitinib, cisplatin, gefitinib, vincristine, vorinostat, and gemcitabine, between the two MMP score groups (Figure 7A). We found that the predicted IC50 values of gefitinib in the low MMP score group were smaller than those in the high MMP score group. In contrast, the predicted IC50 values for sorafenib, sunitinib, vincristine, and vorinostat were relatively lower in the high MMP score group. Aiming at analyzing the efficacy and suitability of immunotherapy and the TME disorder, TIDE score, immune dysfunction, and rejection were taken into full consideration. We found that patients with higher MMP scores tend to have relatively higher TIDE scores, suggesting that patients in the high MMP score group are more prone to immune dysfunction and rejection (Figure 7B).

### Validation of the MMP scoring system using multiple independent datasets

**FIGURE 8:**
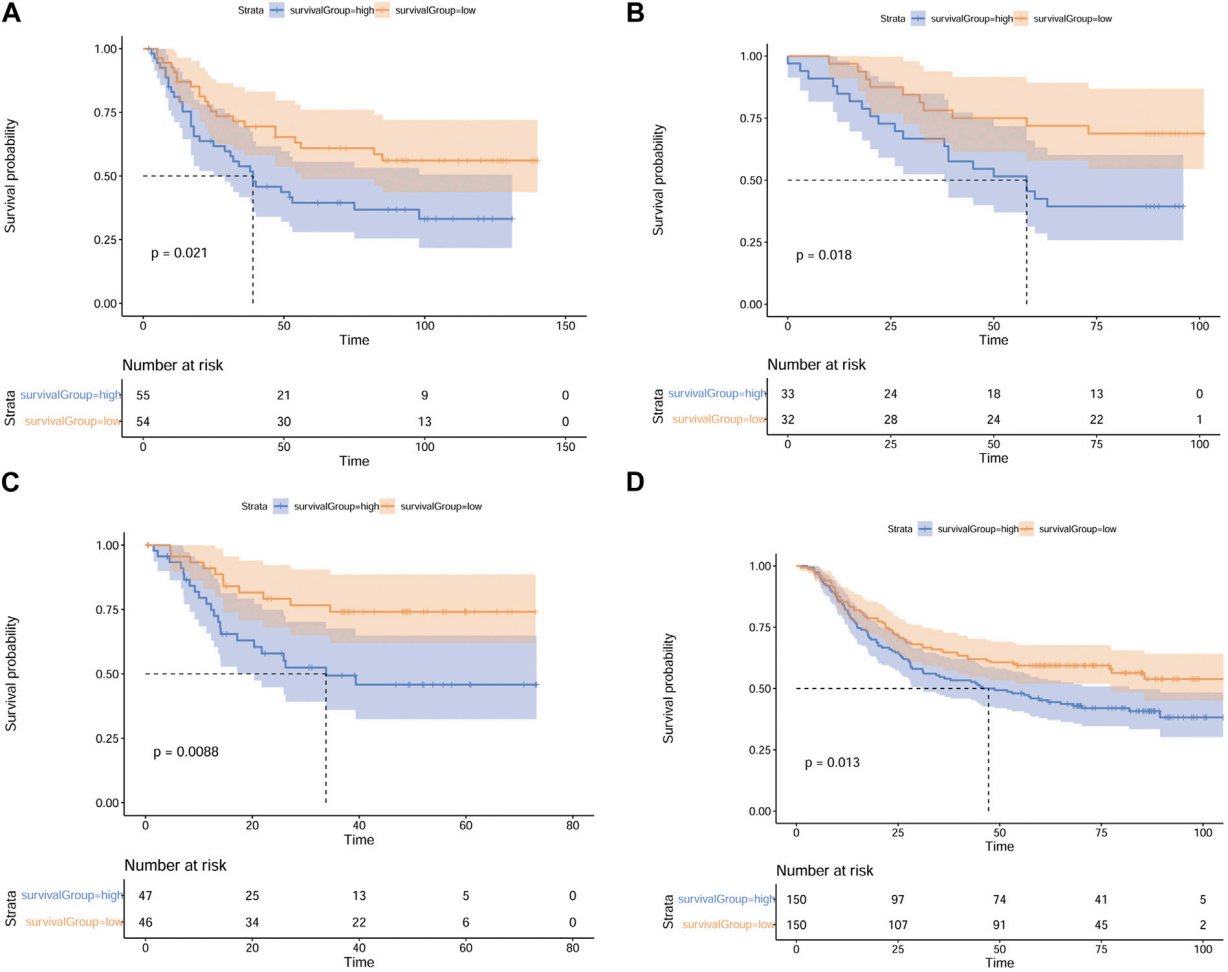
Validation of the MMP scoring system using 4 external datasets. Full validation of the MMP scoring system by four datasets GSE26901. **(A)**, GSE13861 **(B)**, GSE26899 **(C)**, and GSE66229 **(D)** in the GEO database.

To identify the robustness of the MMP scoring system and validate that MMP low scores patients have a more optimistic outcome, we applied the MMP scoring system to four external datasets from the GEO database (GSE26901, GSE13861, GSE26899, and GSE66229) and divided patients in these 4 datasets into high and low MMP score groups. Next, we compared the high and low MMP score groups for each dataset using the log-rank survival analysis; the results are shown in Figures 8A–D. Consistently, these results all show that the OS of patients with GC in the low MMP score group is longer than that of patients in the high MMP score group.

## Discussion

In this study, we systematically and comprehensively analyzed the clinical significance of MMP-related genes and explored their association with tumor microenvironments in patients with GC. We then established an MMP scoring system and explored how the MMP score was associated with prognosis, disease characteristics, and drug treatment effects in patients with GC. Finally, we verified the robustness of the MMP scoring system using external datasets. Since patients with different MMP scores have significantly different clinical traits, genetic mutation characteristics, metabolic characteristics, and drug response characteristics, we believe that clinicians can apply the MMP scoring system to each GC patient for predicting the survival prognosis, and use more effective drugs in the treatment. At the same time, physicians can find more targeted therapeutic drugs and treatment methods through different metabolic pathways and genetic mutations based on the MMP scoring system to improve patients’ prognosis and life quality in the future.

After establishing the MMP-related groups, we compared the survival, MSI scores, and tumor microenvironmental characteristics between the 3 groups. Regarding survival, patients in the C1 group had the best prognoses. Correspondingly, C1 group patients own the highest MSI score. The results above are consistent with previous studies showing that GC patients with high MSI scores have a better prognosis than patients with low MSI gastric cancer of the same pathological type (Baniak et al., 2016; Polom et al., 2018), indicating the robustness and usefulness of the MMP-related groups. The tumor microenvironment plays a pivotal role in tumor progression by interacting with tumor cells. Different studies have demonstrated the inextricable link between tumor-infiltrating lymphocytes and the prognosis of GC (Hu et al., 2019). We found that the TME characteristics of the different MMP-related groups differed. The results of our current research are in line with previous studies showing that the lower the level of CD4^+^ T lymphocyte infiltration, the worse the prognosis of the patient and the more advanced the stage of the tumor would be (Li et al., 2019). Our findings are also supported by a study containing 1524 GC patients that explored the relationship between tumor-derived fibroblasts and clinical features, which showed that higher tumor-derived fibroblast infiltration was associated with a less favorable prognosis (Zeng et al., 2019). Another research, which showed that tumor-associated mononuclear macrophages promote rapid growth and distant metastasis of GC through the secretion of cytokines and growth factors (Qian and Pollard, 2010), is also consistent with our findings. Similarly, a direct correlation between low infiltration of myeloid dendritic cells and good prognoses of malignancy was observed in a large-scale study (Bai et al., 2021), further demonstrating the validity of our MMP-related groups.

A thorough analysis of this MMP scoring system was carried out and we showed that the MMP score could be used as an appropriate indicator for the assessment of the prognosis of GC. This conclusion is supported by the results of other studies exploring the relationship between MMP expression and pathological features of GC. For example, Rybarczyk et al. (2012) found that higher expression of MMP was directly associated with higher infiltration of immune cells and greater lymph node metastasis in GC. Patients in the low MMP score group had higher MSI scores, consistent with a previous study exploring the relationship between MSI and the prognosis of GC (Kim et al., 2017). Because PD-1 inhibitors are more effective for GC patients with high MSI scores (Zhang et al., 2020a), MMP scores have the potential to predict patient response to immunotherapy. Patients with low MMP scores respond better to anti-PD-1 treatment. Immune cells and stromal cells are the main components of the tumor microenvironment, so we used Xcell to estimate the ratios of infiltrated immune cells and stromal cells, and calculate the immune score and stromal score (Yoshihara et al., 2013). High immune scores are significantly associated with high-stage, low differentiation, and poor prognosis of GC (Zeng et al., 2018). This undoubtedly supported our findings. At the same time, we found that low MMP score patients with low stroma scores have better overall survival, consistent with a previous study showing that stroma scores can be used as an independent predictor of prognosis in GC (Mao et al., 2020). Further, based on the stroma score and immune score, a microenvironmental score was constructed by other researchers to compare various features of GC and it was found that a low microenvironmental score predicted a better prognosis of gastric cancer and a lower inflammatory response (Zhou et al., 2017). Correspondingly in our study, the low MMP score patients with better prognoses have the same lower microenvironmental score. In summary, those ramifications demonstrate that the MMP score can be used as a reliable indicator to predict the prognosis of patients with GC.

Studies on the relationship between the tumor stage, tumor differentiation, immune cell infiltration, and prognosis of GC have shown that the more advanced the TNM stage of GC is (Ahn et al., 2010), the worse the differentiation (Wanebo et al., 1993), the less degree of infiltration (Marchet et al., 2008), the more pessimistic the outcome will be. A high MMP score is associated with unpromising clinicopathological characteristics, indicating that the MMP scoring system can be used as an appropriate assessment in clinical practice.

The development and rapid growth of GC and distant metastasis involve a complex array of biological changes. As a result, the impact of MMP as a key factor in GC should be taken seriously. Many researchers have conducted various studies on the biological behaviors of GC through the alteration of different signaling pathways, BP, MF, and CC by MMP. MMP-2 enhances the invasion and metastasis of GC through the NF-KB pathway (Wang et al., 2014b). PPARγ, Cx43, and Grhl2 negatively regulate the expression of MMP-2, thereby inhibiting the progression of GC (He et al., 2007). Silymarin et al. reduced the expression of MMP-2 and MMP-9 by down-regulating the P38MAPK signaling pathway, thereby inhibiting the invasion and metastasis of human GC SGC7901 cells (Lu et al., 2021). In our study, we found that MMP could also alter the biological characteristics of GC through various pathways, implying that MMP-related genes are involved in a wide range of biological alterations. In addition, since the DNA replication, proteasome, DNA strand elongation, as well as histone exchange terms or pathways, were significantly enriched in the low MMP score group, we speculated that these altered biological behaviors might be associated with a good prognosis of GC. Conversely, tyrosine metabolism, calcium signaling pathway, ECM receptor interaction, and immunoglobulin complex terms or pathways may be indicative of a worse prognosis for GC. However, the mechanism of these alterations in signaling pathways or terms (i.e., BP, CC, and MF) affecting the progression of GC remains to be validated.

It is well known that genetic mutations can be involved in the development of gastric cancer by altering several physiological and biochemical processes. Based on the mutations of various genes in the two MMP score groups and the corresponding prognoses, *TTN*, *TP53*, *MUC16*, and *LRP1B* genes might play important roles in influencing the outcomes and clinicopathological features of GC. One study showed that GC patients with wild-type *TP53* had a shorter OS and 1.39 times the risk of death than patients with *TP53* mutations (Deng et al., 2021). Consistently, in our study, most of the patients in the MMP high group contained wild-type *TP53* and their survival prognoses were significantly worse, further supporting the validity and reliability of the MMP scoring system. In a joint study of the relationship between mutations in *MUC4*, *MUC16*, *TTN*, and the prognosis of GC, it was found that the higher the number of mutations in these three genes is, the longer the OS of the patients and correspondingly the higher the TMB (Yang et al., 2020) will be. In addition, a high mutation in *TTN* predicts a good ICB treatment outcome (Jia et al., 2019). Our data also showed that patients in the low MMP score group with a better prognosis tend to have higher *TTN* mutations. What’s more, patients in the low MMP score group with higher MSI scores and lower TIDE scores are likely to have better ICB treatment outcomes, coinciding with the aforementioned study (Jia et al., 2019). After comparing the expression of the *MUC16* gene in GC patients, some researchers concluded that high expression of the *MUC16* gene was significantly associated with a poorer prognosis (Streppel et al., 2012; Jonckheere and Van Seuningen, 2018). While other researchers found that the survival rate of *MUC16* mutant patients was higher than that of MUC16 wild-type patients (Li et al., 2018). Those results are consistent with our study. We speculated that the mutation of *MUC16* could result in a decrease in its expression, thereby improving the prognosis of GC. A gene sequencing analysis of Chinese GC patients showed that *TP53*, *ARID1A*, and *LRP1B* genes were the top 3 genes containing the most mutations (Yu et al., 2021), which is similar to our findings. However, there are no studies investigating the correlation between *LRP1B* gene mutation and the prognosis of GC. Based on the results of our data, we speculated that a high mutation in the *LRP1B* gene might be associated with a good prognosis in GC. Therefore, our data can be used as a reference for the study of the relationship between different gene mutations and GC prognoses. Moreover, it will provide new ideas for screening better prognostic markers for GC in the future.

Identification of new drugs for the treatment of GC has been a clinical priority. The current chemotherapy regimens recommended by the CSCO are SOX, XELOX, and FOLFOX. In clinical practice, however, not all patients benefit from these chemotherapy regimens. Mutations in the *KRAS* and *BRAF* genes, decreased expression of HER-2 in many cases of GC patients, and the fact that bevacizumab is not suitable for most patients, have hindered the application of chemotherapy and targeted therapy in GC. Our study suggests that patients in different MMP score groups might benefit from different drugs. For example, gefitinib is more suitable for patients with low MMP scores, while sorafenib, sunitinib, vincristine, and vorinostat might be more suitable for patients with high MMP scores. Our MMP scoring system could be used to predict drug efficacy, and thus improve the efficacy of chemotherapy or targeted therapy for GC treatment. However, further clinical trials and laboratory validation are still needed. In recent years, immunotherapy has become increasingly popular and it has been elevated from second or even third-line treatment to first-line treatment. To predict and assess the prognosis of ICB treatment, TIDE scores were calculated based on the immune dysfunction of tumor tissue infiltrating CD8^+^ T cells and immune rejection (Jiang et al., 2018). The higher the TIDE score is, the less effective the immunotherapy and the shorter the survival after receiving the treatment will be. Our results imply that immunotherapy might be more appropriate for patients in the low MMP score group, consistent with the aforementioned finding that patients in the low MMP score group with higher MSI scores were more likely to benefit from ICB treatment. This observation further demonstrates the potential of the MMP score to assess and predict the efficacy of ICB treatment in patients with GC. Similarly, we found that immune dysfunction and rejection of tumor-infiltrated cytotoxic T lymphocytes profoundly affect the prognosis of GC. Although patients in the high MMP score group had more CD8^+^ T lymphocytes in their tumor microenvironment, due to the presence of immune dysfunction and rejection, the number of immune cells that are actually effective in eliminating tumor cells is greatly reduced, thus affecting the prognosis of GC.

Finally, the MMP scoring system applied in other GC patients from the GEO database (GSE26901, GSE13861, GSE26899, and GSE66229) validates that the low MMP score group has better prognoses, indicating the robustness, reliability, and usefulness of the MMP scoring system in GC.

Our research initially concludes that MMP influences the progression of GC through the database as well as statistical software analysis, which provides a preliminary verification and framework of the significant role of MMP in the development of GC. In the next step, multiple experiments such as Western blot for exploring the expression of MMP in different stages of GC, MTT assay for elucidating and demonstrating the effectiveness of those 8 drugs mentioned above in inhibiting GC cells, the mechanism of signaling pathways of KEGG and GO affecting GC progression will be carried out to further validate the indispensable role MMP plays in the development of GC.

## Conclusion

In summary, the MMP scoring system proposed in this study provides a comprehensive and integrated assessment of the prognosis, clinicopathological features, tumor microenvironment, and ICB treatment outcomes of patients with GC. There is a significant difference regarding prognosis between the high and low MMP scoring groups. A low MMP score predicts a better prognosis. Therefore, the MMP scoring system could potentially facilitate the diagnosis and personalized treatment of GC patients in clinical practice.

## Materials and methods

RNA sequencing (RNA-seq) profiles and matched complete clinical information (age, sex, survival status, staging, and staging) were retrieved from TCGA (https://www.cancer.gov/tcga) for STAD (n = 411). TCGA is a landmark cancer genomicsprogram, which molecularly characterized over 20,000 primary cancer and matched normal samples spanning 33 cancer types. This joint effort between NCI and the National Human Genome Research Institute began in 2006, bringing together researchers from diverse disciplines and multiple institutions.

### Establishment of MMP-related molecular model group

The Molecular Signatures Database database (https://www.gsea-msigdb.org/gsea/msigdb/index.jsp) is a resource of tens of thousands of annotated gene sets for use with GSEA software, from this website, we can browse, investigate and download gene sets (Subramanian et al., 2005). Two gene sets in the database were selected, where REACTOME_ACTIVATION_OF_MATRIX_METALLOPROTEINASES was contributed by Reactome (https://reactome.org/PathwayBrowser/#/R-HSA-1592389) and WP_MATRIX_METALLOPROTEINASES was contributed by WikiPathways (http://www.wikipathways.org/instance/WP129_r117774). We defined 45 genes in total involved in these two gene sets as MMP-related genes in GC. Then we use the ConsensionClusterPlus package in R software to estimate the number of unsupervised classes in the TCGA-STAD dataset (Wilkerson and Hayes, 2010). The PCA in the ggplot2 package in R software was then applied to downscale the expression profile subsets of the above genes.

### MMP-related groups in GC patients have significant differences regarding prognosis and tumor microenvironment characteristics

Heat maps were plotted for these three MMP-related groups and the survminer package in R software was applied to plot survival curves for OS, PFS, DSS, and DFS and box line plots for MSI scores. MSI score was calculated through MANTIS software, which is a tool for identifying microsatellite instability in paired tumor-normal patient samples (Bonneville et al., 2017; Kautto et al., 2017). Cell-type enrichment analysis was performed on each GC sample by XCell software (https://xcell.ucsf.edu/). XCell is a web tool that performs cell type enrichment analysis from gene expression data for 64 immune and stroma cell types, which uses a novel method that integrates the advantages of gene set enrichment with deconvolution approaches (Aran et al., 2017). The levels of infiltrated immune cells were compared between groups by the Kruskal-Wallis test and visualized with box plots.

### Development of an MMP scoring system for GC patients

The limma package in R was used to identify the DEGs in these three groups (C1, C2, and C3), with thresholds of adjusted *p* < 0.05 and |Log2FC| > 1 (Ritchie et al., 2015). The DEGs positively and negatively correlated with cluster features are named MMP gene signatures A and B, respectively. The dimensionality reduction of MMP gene signatures A and B was achieved using the Boruta algorithm, and the MMP score was calculated according to the following formula: MMP score = ∑PC1A—∑PC1B, where PC1A represents the first component of signature A and PC1B indicates the first component of signature B (Zhang et al., 2020b). The ggalluvial package was used to draw the Alluvial diagram to visualize the three groups regarding MMP scores and survival status (Brunson, 2017). The survminer and survivalROC packages were then used to plot survival curves of OS, PFS, DSS, and DFS and ROC curves for the model. The levels of infiltrated immune cells were then compared between the high and low MMP score groups by the Kruskal-Wallis test. Finally, box plots of MSI scores were plotted for the high and low MMP score groups.

### Association of MMP scores with clinicopathological features of GC

We fused the patients’ clinical data with MMP scores and applied R software to draw box plots depicting the relationship between T, N, M, G, TNM Stages, three MMP-related groups, and MMP scores.

### Gene probe enrichment analysis of DEGs

KEGG and GO enrichment analyses were performed using GSEA software for the high and low MMP groups. Pathways with FDR < 0.05 were considered significantly enriched and enriched signaling pathways were represented using plot bubble maps using the ggPub package. GSEA software uses a computational method that determines whether an *a priori* defined set of genes shows statistically significant, concordant differences between two biological states (Subramanian et al., 2005).

### Gene mutations in different MMP score groups

The mutation data in GC patients were downloaded from the TCGA database. The data were applied to the maftools package to depict the mutation maps for patients in the high and low MMP score groups. Maftools package offers a multitude of analysis and visualization modules that are commonly used in cancer genomic studies, including driver gene identification, pathway, signature, enrichment, and association analyses (Mayakonda et al., 2018). The numbers of mutations were represented using the box plots. The number of mutated genes that differed between the two groups was represented using the Forest plots. *p* < 0.05 was considered statistically significant.

### MMP scores and drug IC50 inference

The database information collated from the pRRophetic package in R was invoked to calculate the IC50 values for the eight drugs, which were visualized using the box plots. In the pRRophetic package, we need only provide the baseline gene expression and IC50, the predicted drug sensitivity is then calculated (Paul G et al., 2014). TIDE stands for Tumor Immune Dysfunction and Exclusion. It is a computational framework developed to evaluate the potential of tumor immune escape from the gene expression profiles of cancer samples. The TIDE score, scores for immune dysfunction and rejection were calculated for both groups of patients using the TIDE website (http://tide.dfci.harvard.edu/login/) and plotted using the box plots (Fu et al., 2020).

### Independent cohort validation of the MMP scoring system

Survival curves for the GC patients in four validation datasets in the GEO database (https://www.ncbi.nlm.nih.gov/geo/) were plotted by applying the survminer package and tested using the Log Rank method. GEO is an international public repository that archives and freely distributes microarray, next-generation sequencing, and other forms of high-throughput functional genomics data submitted by the research community (Kumar et al., 2019). We selected four datasets (GSE26901、GSE13861、GSE26899、GSE66229) about gene expression profiles of GC from GEO for the validation group. (GSE26901 from the Kosin University College of Medicine, GSE13861 from the Yonsei University Severance Hospital, GSE26899 from the Korea University Guro Hospital, and GSE66229 from the Asian Cancer Research Group) (Cho et al., 2011; Oh et al., 2018; Nshizirungu et al., 2021).

## Data Availability

The original contributions presented in the study are included in the article/supplementary material, further inquiries can be directed to the corresponding authors.
